# A past with uncertainty, a future with hope – rare disease day 2014 from a USA perspective

**DOI:** 10.1186/1750-1172-9-31

**Published:** 2014-02-28

**Authors:** Stephen C Groft

**Affiliations:** 1 Office of Rare Diseases Research, National Center for Advancing Translational Sciences, National Institutes of Health, Bethesda, MD 20892-4874, USA

**Keywords:** Rare disease, Orphan product, Translational research resources, Undiagnosed disease, Rare disease day 2014

## Abstract

We reflect on the worldwide research accomplishments, orphan product approvals, and the commitments by the rare diseases community. Major collaborative efforts by the public and private sectors increased interventions and diagnostics for rare diseases. We marvel at the impact of safe and effective treatments when they become available. Hope remains for the rare diseases community with a renewed commitment and acceptance of collaborative public-private partnerships of government translational research and regulatory programs, the biopharmaceutical, devices and diagnostics industries, academic investigators, and patient advocacy groups. The global rare diseases community remains strong and responsive to the voices and needs of patients, families, and clinicians awaiting diagnosis and treatment for their disease. We anticipate even greater successes in 2014 to reflect on Rare Disease Day 2015.

## 

As the celebrations of Rare Disease Day occur throughout the world, we pause to reflect on the numerous and impressive research accomplishments and orphan product approvals granted during the past year. Not to be forgotten are the deep commitments required to reach these goals made by the rare diseases community including the pharmaceutical, biotechnology and medical devices and diagnostic industries, philanthropic foundations, patients and families, medical and scientific advisory boards associated with patient advocacy groups, the academic research community, research and regulatory scientists, government funding and regulatory agencies and the public.

While we marvel at the impact of treatments now available for many rare diseases, the enthusiasm is tempered by the needs of patients and health care providers for interventions to become available for conditions. Despite intensive efforts a lack of an appropriate intervention exists for most rare diseases. The current model of product development for rare disease interventions is extremely expensive and time consuming to complete. Even after the completion of required studies most products are susceptible to failure at different stages of the research and development pipeline. Current estimates suggest nearly 15 years of research and development efforts costing over $1 billion are required before the introduction of a new molecular entity into the marketplace. Even with these significant commitments of time and financial resources, we continue to achieve a 1% success rate of a product reaching the patient. This has resulted in little hope for producing treatments or interventions for most patients with rare diseases.

The identification of new rare diseases is expected to continue to grow and surpass the number of new products available for the prevention, diagnosis, or cure of rare diseases. To meet the prevention, diagnostic and therapeutic needs of these millions of patients worldwide with one of approximately 6500 rare diseases and conditions, a systematic and coordinated approach is still required. The approach will require extensive public-private partnerships to utilize existing resources and incentives to stimulate the development of orphan products. This approach requires not only an acceptance of the premise that there are tremendous unmet health needs for the millions of patients with rare diseases and their families but a renewed commitment to these unmet needs to address this public health problem worldwide.

Despite these drawbacks, there is hope for the rare diseases community. In 2013, a survey of current orphan product development completed by the Pharmaceutical and Research Manufacturers Association (PhRMA) revealed approximately 454 orphan products were in different stages of development. The rare diseases community awaits the arrival of these products into research clinics and in the market place for their use. The Office of Orphan Products Development at the USA FDA provided Orphan Product Designations for more than 255 possible indications. The FDA Review Divisions approved products for 30 rare diseases indications. Regulatory incentives now provide for bestowing a transferable Rare Pediatric Diseases Priority Review Voucher similar to Tropical Diseases Priority Review Voucher for these disorders when an approval for a product for these conditions is granted. Other incentives provide for expedited program development and review include Fast Track Designation, Priority Review status, accelerated approval, special incentives for development of antibiotics for selected diseases, and designation as a breakthrough therapy. Each of these programs includes distinct qualifying criteria and incentives for the community to become more familiar.

The realization exists for most interventions for rare diseases that there is an absence of sufficient pre-clinical and clinical information to enable a potentially useful product to move forward in the research and development continuum. On top of the programmatic and financial commitments to rare diseases research ($3.623 Billion USD for approximately 9400 research projects) and orphan drug research ($809 Million USD for 1650 research projects), the National Institutes of Health (NIH) has initiated new programs and expanded existing programs to provide the collaborative efforts required to stimulate product development or provide necessary data to enable products to move to the next stage of product development for rare, neglected, and common diseases. Several NIH institutes and centers have increased their available resources to bridge these data gaps that currently exist and may provide the traditional grants and contracts to investigators to complete the necessary work. Others will continue the collaborative efforts and provide services and support for the studies to develop the necessary information leading to a regulatory decision. Several of these programs available from the National Center for Advancing Translational Sciences including the competition of the Rare Diseases Clinical Research Network (RDCRN) Consortia, the Therapeutics for Rare and Neglected Diseases (TRND) and the Bridging Interventional Development Gaps Programs, the Drug Repurposing Program, and the Clinical and Translational Sciences Award (CTSA) program complement other models of collaborative efforts leading to product development. These models include the National Cancer Institute’s Experimental Therapeutics program (NExT), and the NeuroNExT program from the National Institute of Neurological Disorders and Stroke, the Best Pharmaceuticals for Children Act (BPCA) at the National Institute of Child Health and Human Development and the Centers for Accelerated Innovation at the National Heart, Lung, and Blood Institute. Each program offers unique opportunities and requires discussions and collaborative efforts with responsible program staff. Growing emphasis on integrating information from existing and newly created patient registries and natural history studies will expand the knowledgebase for a better understanding of rare diseases.

Numerous efforts by the rare diseases community have resulted in an increase of possible interventions and diagnostics for rare diseases. With the ready access and the acceptance of the numerous collaborative approaches and resources available from many sources, more rapid development of products will occur. More recent discoveries of the molecular bases of rare diseases have enabled the identification of many possible therapeutic interventions. With support provided from the Common Fund, the Undiagnosed Diseases Program at the NIH Clinical Center Hospital will expand to more academic centers in the USA as a response to the need for better diagnostic procedures for an estimated 6% to 8% of the population who do not have an adequate diagnosis for their diseases. We anticipate the day when clinics dedicated to the diagnosis of rare diseases will be connected globally. More affordable access to results of sequencing efforts to establish the molecular basis of rare, genetic diseases will provide needed relief to those patients without a diagnosis, even if interventions are not available.

Challenges still remain such as patient recruitment from multiple countries, establishing trust in global research partnerships with appropriate sharing of data and accomplishments, the use of appropriate study design for studies with small patient populations available for a study, and the costs of orphan products.

However, optimism prevails as we consider the possible treatments related to the development of traditional small molecules, enzyme replacement therapies, monoclonal antibodies, gene and cellular therapies, RNAi compounds, nanotechnology applications, and precision or personalized medicines. Establishing and confirming diagnosis will be improved due to whole genome and exome sequencing capabilities. These advances and including many more rare diseases in the International Classification of Diseases (ICD 11) should help establish useful and reliable prevalence figures for rare diseases. Let’s hope that as we celebrate Rare Disease Day 2015 we will look back on Rare Diseases Day 2014 as a continuation of many activities initiated since 1982.

Recognition and acceptance of the expanding role of patient advocacy groups and patients/families as research partners are required for advances with collaborative partnerships including industry, patient advocacy groups, academic research centers, government and foundation funding sources, and regulatory agencies. We can expect a continued expansion of public–private partnerships of government translational research programs with industry partnerships, and industry partnerships with academic research centers and patient advocacy groups on a global basis.

## Competing interests

The author declares that he has no competing interests.

## Author information

Stephen Groft, Pharm.D., retired in February 2014 from his position as Director of the Office of Rare Diseases Research, National Center for Advancing Translational Sciences, at the National Institutes of Health (USA).

